# Non-Coding RNAs in the Regulation of Hippocampal Neurogenesis and Potential Treatment Targets for Related Disorders

**DOI:** 10.3390/biom13010018

**Published:** 2022-12-22

**Authors:** Zhengye Tan, Wen Li, Xiang Cheng, Qing Zhu, Xinhua Zhang

**Affiliations:** 1 Department of Anatomy, Institute of Neurobiology, Medical School, Co-Innovation Center of Neuroregeneration, Nantong University, Nantong 226001, China; 2 School of Pharmacy, Nantong University, Nantong 226001, China; 3 Key Laboratory of Inflammation and Molecular Drug Target of Jiangsu Province, Nantong 226001, China; 4 Central Lab, Yancheng Third People’s Hospital, The Sixth Affiliated Hospital of Nantong University, Yancheng 224001, China

**Keywords:** non-coding RNAs, neurogenesis, neural stem cell, hippocampus, differentiation

## Abstract

Non-coding RNAs (ncRNAs), including miRNAs, lncRNAs, circRNAs, and piRNAs, do not encode proteins. Nonetheless, they have critical roles in a variety of cellular activities—such as development, neurogenesis, degeneration, and the response to injury to the nervous system—via protein translation, RNA splicing, gene activation, silencing, modifications, and editing; thus, they may serve as potential targets for disease treatment. The activity of adult neural stem cells (NSCs) in the subgranular zone of the hippocampal dentate gyrus critically influences hippocampal function, including learning, memory, and emotion. ncRNAs have been shown to be involved in the regulation of hippocampal neurogenesis, including proliferation, differentiation, and migration of NSCs and synapse formation. The interaction among ncRNAs is complex and diverse and has become a major topic within the life science. This review outlines advances in research on the roles of ncRNAs in modulating NSC bioactivity in the hippocampus and discusses their potential applications in the treatment of illnesses affecting the hippocampus.

## 1. Introduction

Non-coding RNAs (ncRNAs) are special RNA transcripts that compose a major portion of the human transcriptome; more than 90% of the human genome is actively transcribed, but only 2% of the entire genome encodes protein-coding RNAs [1,2]. With the exception of several ncRNAs with open reading frames, ncRNAs generally do not encode proteins. Nonetheless, they act as important regulators of development, proliferation, transcription, post-transcriptional modification, apoptosis, cell metabolism, and other biological processes [3]. Transfer RNAs (tRNAs), ribosomal RNA (rRNA), small nuclear RNAs (snRNAs), and ribozymes are all ncRNAs. The structure and function of these non-coding rRNAs have been well documented. Other types of ncRNA include microRNAs (miRNAs), piwi-derived small RNAs (piRNAs), small interfering RNAs (siRNAs), tRNA-derived small RNAs (tsRNAs), long non-coding RNAs (lncRNAs), small nucleolar RNAs (snoRNAs), circular RNAs (circRNAs), and pseudogenes (Ψ). These ncRNAs vary in size, ranging from small to large, as well as in spatial structure, and are usually classified on the basis of their length. Small ncRNAs are less than 200 nucleotides long and include siRNAs, miRNAs, and piRNAs, whereas transcripts longer than 200 nucleotides are termed lncRNAs. Small ncRNAs, such as miRNAs and piRNAs, are <32 nucleotides in length, whereas lncRNAs can be as long as 5000 nucleotides. These RNAs are involved in virtually all cellular processes. Additionally, lncRNAs also regulate gene and protein expression levels through diverse complex mechanisms.

ncRNAs are specifically expressed in different tissues and organs at different stages of development or disease [4]. Many ncRNAs have been found to control transcription and translation, thus ultimately affecting development and disease progression; however, the exact activities of most ncRNAs remain unclear. Moreover, recent studies have indicated that ncRNAs act as key regulators of cell proliferation and death [5]. In recent decades, research on the activity of neural stem cells (NSCs) has focused primarily on niche, molecular, and protein-coding genes. However, studies increasingly indicate that ncRNAs play key regulatory roles in NSC activity. Many factors, including ncRNAs in the adult hippocampus, have been found to affect neuronal differentiation and growth by modulating various signaling pathways [6].

For instance, the expression of miRNAs is highly concentrated in the brain tissues of both humans and rodents and is distributed in several anatomical areas of the brain. For example, hippocampus-specific expression of miR-128a/b, miR-218, miR-138, miR-222, miR-26a, miR-221, and let-7c has been reported [7]. miRNA expression varies by cell type. miR-124 and miR-128 are two examples of miRNAs that are expressed primarily in adult neurons but not in glial cells, whereas miR-23 is found in astrocytes [8] and miR-92b is specifically expressed in NSCs [9]. Furthermore, miR-125b and miR-93 are highly expressed in NSCs of the subventricular zone (SVZ) region [10]. miRNAs have also been implicated in adult neurogenesis.

The hippocampus is essential for complex neurological functions such as learning, memory, and the processing of emotional data. Adult resident NSCs in the subgranular zone (SGZ) undergo proliferation, migration, differentiation, and maturation into granular cells in the hippocampal granular cell layer under the influence of pathological stimuli. They also produce axons that target and form functional synaptic connections with neurons in the CA3 region, integrating into the functional neural loop of the hippocampus [11,12,13]. Hippocampal neurogenesis in the hippocampal dentate gyrus (DG) plays a prominent role in the formation and self-repair of spatial learning and cognitive memory [14,15].

ncRNAs have been increasingly observed participating in hippocampal neurogenesis and functional reconstruction, including NSC regeneration, neural circuit repair, and recovery of learning and memory. This review summarizes progress in the understanding of the roles of ncRNAs in the regulation of hippocampal neurogenesis, particularly the differentiation of NSCs, and describes potential applications in the treatment of hippocampus-associated diseases, thus providing directions for future research and clinical practice.

## 2. Effects of miRNAs on the Differentiation of Hippocampal NSCs

miRNAs are highly conserved ncRNAs approximately 22 nucleotides in length. They are ubiquitous in eukaryotes and have several biological characteristics, including (1) high conservation, (2) temporal expression specificity, and (3) tissue expression specificity. The nucleotide sequence at position 2−8 of the 5′ end of mature miRNA is generally agreed to be the seed sequence (the region where miRNAs specifically bind targeted mRNAs). The roles of miRNAs and their target mRNAs are not one-to-one. A single miRNA usually targets and regulates multiple mRNAs, and the same mRNA can also be targeted and regulated by multiple miRNAs, thus forming a complex overlapping regulatory network. As summarized in Table 1, miRNAs are believed to regulate the differentiation, proliferation, and apoptosis of NSCs. 

### 2.1. miR-132

miR-132, an important miRNA affecting the differentiation of NSCs, is highly expressed in brain-specific DG regions and neurons and was originally observed in the nerve tissues of mice [16], with later observations in humans [17], zebrafish [18], and cows [19]. Studies showed that overexpression of miR-132 promoted the differentiation of NSCs into astrocytes but inhibited neuronal formation [20,21], and miR-132 knockdown impaired integration of newborn neurons into the adult dentate gyrus [22]. The level of miR-132 decreased in the hippocampus during progression of Alzheimer’s disease (AD); this miRNA is required for proliferation of neural precursors and neuronal differentiation in the adult dentate gyrus [23]. The above reports indicate that miR-132 is firmly involved in the progress of hippocampal neurogenesis.

**Figure 1:**
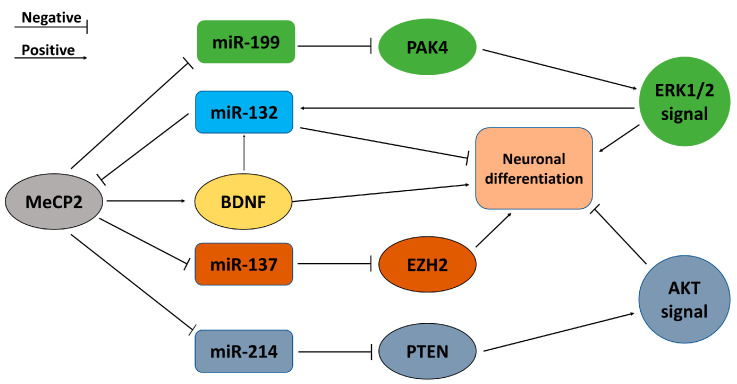
The feedback loops of miRNAs regulated by MeCP2 promoting or repressing neuronal differentiation.

Evidence implies that miR-132 regulates the differentiation of hippocampal NSCs through a variety of actions, particularly in early stages of neuronal development. In early stages of NSC differentiation, miR-132 fine-tunes Notch signaling (very important for the maintenance of undifferentiated NSCs [24]) through its target, Ctbp2, and regulatory pathways involving REST and Sirt1, which inhibit the proliferation of radial glial cells (early stage NSCs) and promote the differentiation of oligodendrocytes [25]. MeCP2 (X-linked methyl-CpG binding protein 2) is a multi-functional epigenetic factor modulating the microenvironment and is critical for normal brain development [26], including hippocampal neurogenesis [27]. MeCP2 is the target of and is regulated by miR132. When miR-132 is overexpressed, it causes simultaneous downregulation of MeCP2 and inversely blocks miR132 mediated-increases in MeCP2 [20,28,29]. miR132 itself was induced by BDNF, a neurotransmitter modulator whose expression was in turn controlled by MeCP2, while the loss of MeCP2 reduced BDNF and miR132 levels in vivo [29]. Obviously, MeCP2-BDNF-miR132 forms a regulatory feedback loop compensating the homeostasis regulatory network in the brain to ensure microenvironmental balance (as shown in Figure 1). ERK1/2 signaling is crucial for neuronal proliferation and differentiation, as well as neurite outgrowth. Moreover, blocking the ERK1/2 signaling pathway decreases miR-132 levels and neuronal differentiation. This signaling pathway therefore controls miR-132 production—a crucial step in the early neuronal lineage of stem cells [30]. The reports above signified the complicated mechanisms mediating the role of miR-132 controlling neurogenesis.

### 2.2. miR-199 and mir-214

Mellios and colleagues found that miR-199 and miR-214 are strongly upregulated in patients with Rett syndrome and MeCP2-deficient neuronal progenitor cells (NPCs), and also found that they are important effectors in early neuronal development through multiple effects on the AKT and ERK signaling pathways [31]. In MeCP2-deficient NPCs, upregulated miR-199 and miR-214 have different regulatory effects on the ERK and AKT signaling pathways. The upregulation of miR-214 decreases the expression of PTEN, whereas the upregulation of miR-199 significantly inhibits the expression of PAK4 in MeCP2-deficient neurons. In the hippocampus of MeCP2-deficient mice, miR-199/214 is upregulated and expression of targets PAK4 and PTEN decreases, thus resulting in differential regulation of upstream ERK and AKT signaling pathways in neurogenesis on the basis of inactivation of the ERK pathway and activation of the AKT pathway. Moreover, changes in the expression of miR-214/PTEN and miR-199/PAK4 in 3-week-differentiated neurons derived from iPSCs led to both ERK and AKT activation, thus indicating a compensatory effect on the activation of the two central molecular centers [31]. As shown in Figure 1, miR-132, MeCP2, miR-199/214, and the ERK and AKT pathways form a regulatory loop modulating the differentiation of hippocampal NSCs.

### 2.3. miR-140-5p

According to previous research, miR-140-5p is highly expressed in patients with post-stroke depression, and its expression positively correlates with depression severity [32]. miR-140-5p was likewise upregulated in a mouse model of bilateral common carotid artery ligation, and consequently may limit hippocampal neurogenesis and exacerbate cognitive impairment [33]. 

miR-140-5p has been confirmed to target and downregulate expression of Prospero Homeobox 1 (Prox1), which mediates the inhibition of hippocampal neurogenesis [33,34]. Prox1 is found in the thalamus, cortex, hypothalamus, DG, and cerebellum during the prenatal, postnatal, and adult development phases [35,36,37]. Under the influence of Wnt signaling, the development of nascent neurons in the granular layer of the adult hippocampus is regulated and controlled by Prox1 [38]. Additionally, Prox1 facilitates NSC differentiation and cell cycle withdrawal by inhibiting the Notch pathway [39]. 

Prox1 is essential in the differentiation and maturation of glutamatergic central neurons and intermediate progenitors during hippocampal neurogenesis in adults [38,40,41]. In addition, the staining of GFAP, DCX, and MAP-2 during miR-140-5p overexpression has indicated that miR-140-5p inhibits neuronal differentiation of NSCs but promotes astrocytic differentiation via the production of synapse-associated proteins. The ERK/MAPK signaling pathway is stimulated by miR-140-5p through negative regulation of the downstream target Prox1 [34]. In conclusion, miR-140-5p inhibited neuronal differentiation via negatively regulating Prox1 and activating the ERK/MAPK signaling pathway.

### 2.4. miR-137

miR-137 is enriched in both embryonic and adult brains and has been reported to promote adult SVZ NSC differentiation into neurons and to inhibit their maturation in the adult hippocampus [42,43]. The gene encoding this miRNA has been confirmed to be located on human chromosome 1p22 within the non-protein-coding gene BRAMY2014205 [44], and has also been observed regulating the expression of more than 1000 predicted target genes [45,46]. Therefore, identifying the cascades involving miR-137 and the fate of NSCs is of great importance.

Functional experiments have confirmed that miR-137 is involved in neuronal development, and studies in a mouse model revealed that miR-137 suppresses cellular proliferation while facilitating neurodevelopment. Forced expression of miR-137 in mouse embryonic NSCs decreases proliferation but enhances premature neuronal differentiation [42]. Moreover, NSCs within the SVZ display similar effects [43]. Recently, Channakkar et al. found that miR-137 overexpression in human iPSC-derived NSCs (hiNSCs) significantly increases newly formed neurons [47]. However, miR-137 exhibits different effects on adult hippocampal neurogenesis in the dentate gyrus. Overexpression of miR-137 disrupts neuronal differentiation as well as dendritic arborization [48]. Szulwach et al. also found that miR-137 overexpression inhibits maturation and promotes proliferation of adult NSCs, whereas antagonism of miR-137 promotes the differentiation of neurons and decreases proliferation [46]. This apparent disparity is probably due to the diverse ways in which miR-137 participates in various phases of neuronal development, as well as the inherent characteristics of cells in various neurogenic zones. These studies have demonstrated that correct neuronal differentiation, proliferation, and modulation of neurogenesis depends on balanced expression of miR-137.

The mechanism mediating the effects of miR-137 on the neuronal differentiation of NSCs may be related to the feed-forward self-regulating loop between miR-137 and OCT4 or SOX2 [47]. OCT4 and SOX2 are both pluripotent transcription factors with key roles in stem cell maintenance and differentiation [49,50,51]. Boyer et al. found that OCT4 and SOX2 co-occupy the promoter of mir-137 [51]. Direct binding to the 2.5 kb upstream region of miR-137 with MeCP2 and Sox2 in adult NSCs and H3-K4-Mono-Me and H3-K9-Tri-Me epigenetic modification induced by MeCP2 caused downregulation of miR-137 expression targeting Ezh2, a histone methyltransferase, thereby increasing proliferation and decreasing neuronal differentiation [46]. However, Channakkar et al. found that miR-137 overexpression in hNSCs increases OCT4 and SOX2 expression [47]. Therefore, miR-137 and OCT4 or SOX2 have been suggested to form a feed-forward self-regulating loop involving OCT4 and SOX2 as key components [51,52], through which miR-137 correctly regulates neurogenesis. 

### 2.5. miR-153

miR-153 is highly conserved in zebrafish and humans and is highly expressed in the hippocampus in young rats and infants; it additionally plays a major role in adult neurogenesis [53,54]. According to a study by Qiao et al., endogenous miR-153 expression is downregulated with decreasing differentiation of neurons and is reduced with increasing numbers of NSC passages in vitro [55] However, the number of developed neurons has been found to increase after forced miR-153 expression in late NSCs, thus suggesting that miR-153 promotes neuronal differentiation.

Additionally, miR-153 has been found to control the Notch signaling pathway by preventing the translation of Jagged1 and Hey2. In vivo results demonstrated that overexpression of miR-153 in the hippocampus of old mice increased neurogenesis and cognitive impairment [55], implying the potential target for treatment.

### 2.6. miR-335-3p

miR-335-3p, whose gene is located on chromosome 7q32.2, is crucial in the emergence of a variety of cancers and tumors. This miRNA functions as a critical regulator of cell fate [56]. Furthermore, miR-335-3p is involved in a multitude of malignant tumors of the central nervous system (CNS), including well-known diseases such as astrocytoma and glioma [57]. Additionally, miR-335-3p specifically targets FMRP Translational Regulator 1 (Fmr1) [58], which synchronizes the migration of cortical neurons with the growth of the brain in developing mice [59]. In addition, evidence indicates that knockout of Fmr1 in adult NSCs decreases the expression of p53 [60], whereas the p53 signaling pathway promotes the self-renewal of NSCs [61].

As an important transcriptional factor, FoxM1 markedly influences cell proliferation and consequently controls neuroblastoma cell differentiation [62]. Jia et al. found that FoxM1 regulates miR-335-3p expression, as gain or loss of FoxM1 expression respectively increased or decreased miR-335-3p levels [63]. The expression of miR-335-3p also increased after restoration of FoxM1 expression in cells with FoxM1 knockdown. Data suggest that FoxM1 positively controls miR-335-3p expression. Notably, stimulation of the p53 signaling pathway via FoxM1-mediated stimulation of miR-335-3p may inhibit NSC differentiation and promote NSC self-renewal. The expression of FoxM1 in NSCs substantially decreases with increasing differentiation, as demonstrated by qPCR and Western blot analysis; these findings are compatible with the observed decline in the expression of miR-335-3p. According to the aforementioned findings, miR-335-3p is initially activated by FoxM1 and decreases the expression of Fmr1 before activating the p53 signaling pathway, thus increasing NSC self-renewal and preventing differentiation.

### 2.7. miR-410-3p

Accumulating evidence from animal studies has demonstrated that anesthetic exposure affects long term neurocognitive function in the developing brain [64,65,66]. Early research indicated that exposure to sevoflurane in the second trimester of pregnancy inhibits NSC development and triggers early death [67,68,69]. This mechanism may involve miR-410-3p, which is easily influenced by sevoflurane.

An uncommon neurodegenerative condition termed dentatorubral and pallidoluysian atrophy can be caused by mutations in the ATN1 gene. Atrophin-1 (ATN1) is extensively expressed in brain tissues [70] and recently has been suggested to be crucial for the maintenance of NSCs [71]. According to a report by Zhang, frequent exposure to sevoflurane may upregulate the expression of miR-410-3p and downregulate the expression of ATN1 [72]. Double luciferase reporter assays have revealed that ATN1 is the direct target of miR-410-3p.

Primary hippocampal NSCs harvested from the middle period in pregnant rats have been used to study the effects of sevoflurane on the differentiation of hippocampal NSCs. Repeated sevoflurane exposure has been found to give rise to the early differentiation of NSCs and the proliferation of astrocytes in the brains of developing offspring, thus resulting in a long-term decrease in hippocampal neurons. Lentivirus-mediated overexpression of ATN1 may diminish the effects of sevoflurane, and miR-410-3p inhibition via lentivirus transfection might improve NSC differentiation and ATN1 expression, thus indicating that ATN1 mediates the effects of miR-410-3p on hippocampal neurogenesis during sevoflurane exposure.

### 2.8. miR-145

A novel role of miR-145 in the neuronal differentiation of NSCs, as reported by Morgado et al., is inhibition of the self-renewal and pluripotency of human ESCs through the Sox2-Lin28/let-7 signaling pathway network [73]. Sox2, the core transcription factor of ESCs, is necessary for the early differentiation of NSCs, and its knockout can disrupt the differentiation of neurons [74,75]. A delicate balance of Sox2 levels is essential for normal neuron development. A previous report from Cimadamore et al. showed that Sox2 promotes NSC proliferation through Lin28/let-7b miRNA [74], and Sox2 is essential for the maintenance of optimal levels of Lin28, a developmental regulatory RNA binding protein that selectively inhibits the expression of let-7b miRNA. Lin28 and let-7b substantially mediate the function of Sox2 in the proliferation of NSCs.

Morgado et al. reported that Sox2 and Lin28 are direct targets of miR-145, and Sox2, Lin28, and let-7b are all regulated by miR-145, thus forming a double negative feedback loop [73]. However, miR-145 directly downregulates Sox2, thus further decreasing the expression of Lin28. Moreover, miR-145 directly decreases Lin28 expression. The low level of Lin28 results in an increase in let-7b. The above results have indicated that, during neurogenesis, the miR-145/Sox2-Lin28/let-7 signaling pathway might affect the differentiation of NSCs.

### 2.9. Other miRNAs

Previous studies have demonstrated that NSCs transplanted into the SGZ of hippocampi after denervation surgery exhibit robust neuronal differentiation and proliferation [76], implying that the microenvironmental changes in the denervated hippocampus facilitated neurogenesis. The following research revealed ncRNA expression patterns and neurogenesis-associated miRNAs in local exosomes using RNA sequencing and bioinformatic analysis of exosomes. The expression of miR-6324 and miR-3559-3p is notably higher in brains from the ectoderm than in tissue from other organs and is particularly high in the hippocampus. Additionally, overexpression of miRNAs miR-6324 and miR-3559-3p in hippocampal NSCs promotes neuronal differentiation while suppressing proliferation [77]. 

Through online database analyses and RNA-seq, we have also found several miRNAs that affect the proliferation and differentiation of NSCs, including miR-103-3p, miR-130-3p, and miR-107-3p. Reports have indicated that miR-103-3p controls NSC proliferation, differentiation, and apoptosis. Moreover, Ndel1 has been confirmed to be a novel miR-103-3p target whose overexpression induces NSC differentiation into neurons via the Wnt/β-catenin pathway, thus increasing NSC proliferation and decreasing NSC death [78]. These findings suggest a role of miR-103-3p/Ndel1 in NSC biological processes. By controlling the AKT/PI3K pathway, Acsl4 is targeted by miR-130a-3p to mediate the neuronal differentiation of NSCs [79]. Similarly, forced expression of miR-107-3p suppresses the neuronal differentiation of NSCs [80]. These findings have enabled deeper understanding of neurogenesis and might aid in the development of novel therapies for CNS diseases.

**Table 1:** Effects and mechanisms of various microRNAs in NSCs.

Name	Biological Function	Target	References
miR-124	Inhibit apoptosis of NSCs/NPCs Promote survival of hippocampal granule neurons and the normal development of axons	BCL2L13 Lhx2	[81]
miR-132	Inhibit neuronal differentiation of ESs Inhibit proliferation of radial glial cells and promote oligodendrocytes to differentiate	MECP2 P250GAP	[21,82]
miR-145	Inhibit NSCs from differentiating	SOX2	[73]
let-7f	Promote NSCs to differentiate into astrocytes and neurons	Lin28 Colin	[83]
miR-410-3p	Early differentiation of developing offspring brain NSCs	ATN1	[72]
miR-335-3p	Maintain self-renewal and inhibit NSCs from differentiating	Fmr1	[63]
miR-140-5p	Inhibit NSCs from differentiating into neurons Promote differentiation into astrocytes	PROX1	[34]
miR-137	Promote neuronal differentiation of hiNSCs	MEF2A OCT4	[47]
miR-9	Inhibit proliferation of NSCs and promote neuronal differentiation of adult NSCs	TLX	[84]
let-7b	Inhibit proliferation of NSCs and promote differentiation of adult NSCs	TLX cyclinD1	[85]
miR-137	Promote proliferation of NSCs and inhibit neuronal differentiation of adult NSCs Inhibit dendritic growth and the formation of dendritic spines in adult hippocampal newborn neurons	Ezh2 Mib-1	[52]
miR-184	Drive symmetrical division of aNSCs and promote the self-renewal and proliferation of aNSCs	Numb1	[86]
miR-25	Promote the proliferation of aNSCs	FoxO3	[87]
miR-17-92 cluster	Promote proliferation of adult NSCs and promote newborn neurons to differentiate	Sgk1	[88]
miR-195	Promote proliferation of adult NSCs and inhibit neurons from differentiating	MBD1	[89]
miR-19	Promote migration of newborn neurons in the adult hippocampus	RapGEF2	[90]
miR-I5a	Inhibit dendritic maturation of newborn neurons in the adult hippocampus	BDNF	[91]
miR-153	Promote neurogenesis and cognitive ability	Hey2, Jagged1	[55]
miR-199 and miR-214	Promote neuronal differentiation and induce neuronal progenitor proliferation and survival in prenatal development	PAK4, PTEN	[31]

## 3. Regulation of lncRNAs and the Differentiation of Hippocampal NSCs

lncRNAs are defined as a class of RNA molecules that do not encode proteins and whose transcripts are longer than 200 nucleotides. lncRNAs usually have the following characteristics: (1) lncRNA sequences are usually long and have an mRNA-like structure. (2) lncRNAs are not highly conserved, but the local sequences, and consequently their secondary and tertiary structures, are conserved; thus. lncRNAs are functionally conserved. (3) Expression of lncRNAs is relatively low. (4) Most lncRNAs show clear spatial–temporal expression specificity during differentiation and development. (5) Unlike miRNAs, lncRNAs do not have a universal mode of interaction. They can bind RNAs, DNAs, and proteins, and this binding can be enhanced or inhibited [92].

lncRNAs influence gene expression at the epigenetic, transcriptional, and post-transcriptional levels, and consequently are involved in various cellular processes, including cell division, survival, apoptosis, and motility [93]. Their localization enables diverse functions. For example, lncRNAs can function as miRNA “sponges” in the cytoplasm by binding miRNAs and releasing them from their 3′UTR binding sites; consequently, mRNA stability is modulated, and miRNA activity and translation in the nucleus are inhibited. [94,95]. Nuclear lncRNAs regulate chromatin and gene transcription [96]. The regulation of lncRNAs by transcriptional variable splicing is currently unclear, and its relationship to the regulation of neural differentiation requires further study. Table 2 summarized the lncRNAs reported to be related with neurogenesis.

### 3.1. Rik-201 and Rik-203

The lncRNA C130071C03Rik (mouse homologue of LINC00461) contains five transcripts (splice variants), among which the lncRNAs Riken (Rik)-201 and Rik-203 control neural differentiation. The lncRNA C130071C03Rik has been reported to be specifically expressed in the ependymal region of the spinal cord at embryonic days 11.5 and 13.5 in mice, and has been found to be expressed at higher levels in neural tissues than other tissues [97]. In comparison to other tissues, the hippocampus has an elevated level of Rik-203, which increases throughout neural development [98]. Recently, Zhang et al. found that Rik-203 and Rik-201 are present at high levels in the brain and promote mESC differentiation into neurons [99]. In addition, through RNA pull-down experiments, miR-467a-3p and miR-96 have been found to bind Rik-203 and Rik-201, respectively, thus indicating that Rik-203 and Rik-201 may serve as competing endogenous RNAs that inactivate miR-467a-3p and miR-96, respectively. Analysis using online miRNA target predication tools has indicated that Sox6 is a target of both miR-467a-3p and miR-96, and luciferase reporter assays have indicated that miR-467a-3p and miR-96 bind and downregulate Sox6 expression. Sox6 specifically regulates dopamine neurons during neural development and plays a regulatory role in the early differentiation of hippocampal NSCs [100,101]. Furthermore, gain or loss of function experiments have shown that Rik-203 and Rik-201 knockdown and overexpression of miR-96 and miR-467a-3p both inhibit neural differentiation. Sox6 reverses the suppression of differentiation in NSCs by upregulating miR-96 and miR-467a-3p and downregulating Rik-203 and Rik-201. These results indicate that Rik-203 and Rik-201 act as competing endogenous RNAs in the functional inhibition of miR-467a-3p and miR-96, respectively, and modulate the expression of Sox6, thereby further regulating NSC differentiation [99].

Sevoflurane has been extensively used in clinical anesthesia, and early childhood exposure to sevoflurane has been associated with neural abnormalities in both humans and animals according to prior research [98,102]. Sevoflurane exposure potently inhibits NSC self-renewal and differentiation in vitro [103], thus leading to neuronal loss and cognitive impairment in young animals. Sevoflurane has also been found to decrease Rik-203 levels in mouse hippocampal tissue and neural progenitor cells. Suppression of Rik-203 levels in neural progenitors decreases not only Sox1 and Nestin expression, but also the number of Sox1 positive cells. miR-101a-3p strongly binds Rik-203, as evidenced by RNA-RNA pull-down experiments. Sevoflurane, Rik-203 suppression, and miR-101a-3p overexpression all decrease GSK-3β levels; consequently, a cascade of miR-101a-3p and GSK-3β may be involved in the Rik-203-mediated regulation of neural development [98]. Rik-203 also attenuates neuronal differentiation via inhibition of downstream miR-466l-3p. Sevoflurane decreases Rik-203 levels, thereby resulting in the release of miR-466L-3p from Rik-203. When miR-466L-3p is released, it specifically targets and decreases the concentration of BDNF and subsequently inhibits neuronal development [104]. The results above suggest that Rik-203 is a potential target that may be important in the prevention of anesthesia-induced neurotoxicity.

### 3.2. Peg13

The lncRNA Peg13 was first reported to be differentially expressed in cerebral vascular endothelial cells in an ischemic glucose deficiency model in 2016, thus suggesting its potential role in nervous system injury [105]. Recently, Peg13 has been shown to regulate social and sexual interactions in mice, and mice lacking Peg13 exhibit same-sex attraction-like behavior. These mice also exhibit a deficiency in pup retrieval behavior, elevated anxiety, and diminished activity and curiosity [106]. Peg13 has been found to be involved in controlling mouse mating choices and modulating sevoflurane-associated neurotoxicity against NSCs [107,108]. Reports have indicated that an axis between miRNAs and target genes mediating Peg13 sponges is involved in the regulation of biological function. Exposure to sevoflurane significantly downregulates the expression of Peg13 and Sox13 and upregulates the expression of miR-128-3p in neural stem cells. Peg13 serves as a molecular sponge for miR-128-3p, thereby maintaining the expression of Sox13 in NSCs and decreasing sevoflurane-associated neurotoxicity [103]. Peg13 bound to miR-490-3p upregulates Psmd11, thus inactivating the Wnt/β-catenin pathway and alleviating the progression of epilepsy [107]. Mechanistically, Peg13 may function as a sponge for miR-20a-5p, thereby increasing the expression of XIAP and lessening serious brain injury caused by hypoxia/ischemia in newborn mice [109]. The Peg13/miRNA/target gene axis may have promising applications as a potential therapeutic target in neurological diseases.

### 3.3. lncRNA1230

lncRNA1230 is a long intergenic non-coding RNA. Large-scale microarray data from Guttman et al. [107] indicate that the expression of lncRNA1230 is associated with differentiation of the neuroectoderm in mouse embryonic stem cells, thus suggesting its involvement in the control of neural lineage determination. Forced ectopic expression of lncRNA1230 significantly attenuates the ability of mouse embryonic stems to form neural cells, whereas knockdown of lncRNA1230 promotes the conversion of mouse embryonic stem cells toward certain NPCs. The lncRNA1230-induced mechanism of inhibition in the transformation of mouse ESCs to NPCs involves decreasing trimethylation of histone 3 lysine 4, thus inhibiting the binding of WD repeat domain 5 (WDR5) to the promoter regions of neurogenesis-related genes [108]. Notably, lncRNA1230 may play a crucial role in the neural fate of stem cells.

### 3.4. Pnky

Pnky is a highly conserved neuro-specific lncRNA localized primarily in the nucleus. In 2015, Ramos et al. [110] found that Pnky knockdown accelerates the differentiation of NSCs into mature neurons and significantly decreases the numbers of NSCs. Therefore, Pnky plays a critical regulatory role in NSC differentiation. Reports have indicated that Pnky inhibits the differentiation of NSCs by binding and interacting with polypyrimidine bundle binding protein (polypyrimidine tract-binding protein, PTBP1) and consequently inhibiting the splicing and expression patterns of key mRNAs in neuronal stem cells [111]. Pnky has also been shown to be a key regulator of NSC migration through modulation of the splicing and export of target mRNAs [112].

Although lncRNAs play important roles in cell biology, few have been shown to regulate in vivo development, particularly cis and trans-regulation. In one example, after knockout of Pnky in developing cortical cells, the expression of POU Class 3 Homeobox 2 (Pou3f2) decreased and differentiation of NSCs was promoted, although the expression of Pou3f2 was not completely lost with the deletion of the Pnky gene. Pnky promotes the expression of Pou3f2 and regulates the differentiation of NSCs through trans-regulatory mechanisms [113].

In-depth knowledge of Pnky may potentially allow for its use in the treatment of neural disorders. Lin et al. [114] designed and synthesized an MRI visualization nano-drug that immobilizes a mixture of siRNAs targeting Pnky on micelle surfaces and promotes the directional differentiation of NSCs into neurons by downregulating the level of Pnky, thus repairing cerebral infarctions. These results indicate the great potential of nanomedicines targeting Pnky in NSC-based therapies, particularly for stroke.

### 3.5. Neat1

The overexpression and knockdown of the lncRNA Neat1 respectively promotes and inhibits both spinal cord NSC migration and differentiation into neurons [115]. Neat1 has been reported to be the key regulator of Wnt/β-catenin [116], the key pathway affecting the proliferation of NSCs [117]. The expression of Neat1 is regulated by miR-124, thus resulting in activation of the Wnt/β-catenin pathway during spinal cord injury regeneration [115]. Recent studies have indicated that the lncRNA Neat1 mediates the proliferation of neural stem cells via the Neat1-let 7 b-P21 axis [118]. Neat1 may also mediate several pathways regulating neurogenesis. Beyond promoting NSCs to differentiate into neurons, Neat1 promotes NSCs to differentiate into oligodendrocytes. In 2019, Katsel et al. found the diminished expression of Neat1 in the brains of patients with schizophrenia, with a greatly diminished number of oligodendrocytes also thus observed [119]. In terms of the above reports, there exists a feedback loop between lncRNA and miRNA, forming a mutual regulation relationship. As a competing endogenous RNA, miR-124 is the target of Neat1 and conversely controls its expression, and together they regulate downstream gene expression and signal activity.

### 3.6. GAS5

The lncRNA GAS5 has been extensively studied in tumors and is thought to potentially function as an anti-oncogene [120]. Although research on the importance of GAS5 in neurological disorders such as AD is scarce, GAS5 levels are significantly higher in patients with AD than controls [121]. The function of GAS5 in neurogenesis has also been investigated, with GAS5 being found to promote hippocampal NSC differentiation into neurons. In rats with cholinergic injury, overexpression of GAS5 enhances learning and memory in vivo [122]. GAS5 was found to be regulated by Lhx8, which has a specific effect on the development of the cholinergic nervous system and promotes cholinergic differentiation of hippocampal NSCs. Therefore, it has been hypothesized that an elevated GAS5 level in the hippocampus promotes NSCs in the hippocampal DG to differentiate into neurons.

**Table 2:** Effects and mechanisms of various lncRNAs on NSCs.

Name	Biological Function	Mechanism	References
Sox2OT	Inhibit NSC proliferation and promote neuronal differentiation	Link with Sox2, interact with YY1	[123]
RMST	Promote the formation and development of nerve cells	Target Sox2	[124,125]
Kdm2b	Promote the formation and development of nerve cells	Combine with hnRNPAB and activate Kdm2b expression	[126]
Paupar	Promote the formation and development of nerve cells	Combine with local genes Pax6 and KAP1	[127]
Gm21284	Inhibit NSC proliferation while promoting NSC differentiation	Interact with miR-30e-3p, miR-147, and miR-431	[128]
1604	Enhance neural differentiation	miR-200c/ZEB1/2 axis	[129]
Rik-201	Promote neural differentiation	Regulated by C/EBPβ and target miR-96/Sox6	[99]
Rik-203	Promote neural differentiation	Regulated by C/EBPβ and target miR-467a-3p/Sox6 and miR-101-3a/GSK-3β	[98,99]
Malat1	Enhance neural differentiation	Activate ERK/MAPK, inhibit PPAR/p53	[130]
Pnky	Inhibit neural differentiation and the formation and development of nerve cells	Interact with PTBP1	[110]
IncR492	Inhibit neural differentiation	Interact with HuR and activate Wnt signaling	[131]
BDNF-AS	Inhibit eNSC-derived neurite outgrowth	Target TrkB signaling pathway	[132]
UCA1	Promote NSC differentiation to astrocytes	miR-1/Hes1	[133]
OPC	Boost oligodendrogenesis	Regulated by OLIG2	[134]
IncOL1	Boost oligodendrogenesis	Form a complex with Suz12	[135]
Inc158	Boost oligodendrogenesis	Promote NFIB expression	[136]
Neat1	Boost oligodendrogenesis	Activate the Wnt/β-catenin pathways	[115]
Pcdh17it	Function as an oligodendrogenesis marker	Unknown	[137]
OLMALIN/-AS	Modulate oligodendrocyte maturation	May affect several genes, Target multiple genes, such as HDAC9, SOX4, GPR126 and EGR1	[138]
MAG3	Inhibit neurogenesis	Affect Notch or Wnt/β-catenin signaling pathway, miR-128-3p/ATRA/cAMP/CREB axis	[139,140]
lncRNA1230	Attenuate NSCs’ ability to form neural cells	Interact with Wdr5	[141]
LINGO-1	Promote neurogenesis	Downregulate miR-15b-3p and promote Wnt5a expression	[142]
Peg13	Promote neurogenesis	Sponge microRNA-128-3p to preserve Sox13 expression	[103]
GAS5	Promote hippocampal NSC differentiation into neurons	Unknown	[121,122]

## 4. Regulation of circRNAs in Hippocampal NSCs

circRNAs are a newly described RNA species formed by the reverse splicing of linear genes. These stable closed circular RNA molecules are widely conserved and expressed in eukaryotes [143]. circRNAs act as molecular “sponges” for miRNAs and regulate gene transcription, affect protein synthesis and function, and participate in the regulation of intercellular signaling pathways [144]. Clustering of circular RNA sequencing data from different regions of the mouse brain (olfactory bulb, prefrontal cortex, hippocampus, and cerebellum) has indicated that expression of these RNAs is brain region-specific [145], and circRNAs are highly correlated with CNS diseases [146,147].

lncRNAs, miRNAs, and circRNAs are inextricably linked; for example, circRNAs and lncRNAs can negatively regulate the expression of miRNA through an miRNA sponge mechanism, and can further interfere with the complementary base pairs of target mRNAs [148], thus modulating gene expression and interfering with the occurrence and development of disease [149]. Many ncRNAs do not play independent roles in cells but mutually interact and collaboratively influence biological functions. Table 3 summarized the circRNAs reported to be related with NSCs.

### 4.1. circHIPK2

Wang et al. showed that circHIPK2 plays a negative role in the neural differentiation of NSCs. In vitro silencing of circHIPK2 causes direct differentiation of NSCs to neurons but not to astrocytes. Transplantation of NSCs with downregulated circHIPK2 has been found to facilitate neural plasticity and functional recovery after stroke; therefore, circHIPK2 may be a potential target for stroke treatment [150]. 

RNA sequencing has indicated that circHIPK2 targets and increases the expression of Smox. Silencing any of them facilitates neuronal differentiation in vitro but does not affect differentiation into astrocytes [150]. Thus, the effect on neuronal differentiation after silencing of circHIPK2 is potentially mediated by inhibition of the expression of Smox, thereby further promoting nerve recovery after stroke. However, the identities of the intermediary molecules between circHIPK2 and Smox remain elusive.

miR-124 was proposed to have an intermediary role after Huang et al. discovered that circHIPK2 functions as an endogenous sponge for miRNA-124—a miRNA that regulates autophagy and endoplasmic reticulum stress [146]. Interestingly, miRNA-124 is involved in the regulation of neural differentiation [151,152], and Smox has been found to be the target gene of miRNA-124 in the development of H. pylori-associated gastric cancer [153]. The circHIPK2/miR-124/Smox axis is believed to play an important role in neurogenesis, although this possibility has not been confirmed experimentally.

### 4.2. circ-TTC3

circRNA tetratricopeptide repeat domain 3 (circ-TTC3) has been studied in a limited number of diseases but is known to be upregulated in hypoxic cardiomyocytes and to protect against myocardial infarction-induced cardiomyocyte apoptosis via the miR-15b-Arl2 regulatory cascade [154]. Additionally, circ-TTC3 binds miR-449a, thus activating the NFⱪ-B and PI3K/AKT pathways and ameliorating hypoxia-induced damage in HaCaT cells [155]. Moreover, during acute kidney injury caused by sepsis, circ-TTC3 alleviates inflammation and oxidative stress via the miR-148a/Rcan2 axis [156].

The effect of circ-TTC3 on NSC differentiation has been demonstrated by Yang et al. [157]: depletion of circ-TTC3 in NSCs increases proliferation and neuronal differentiation. circ-TTC3 appears to sponge miR-372-3p and then target and enhance Toll-like receptor 4 (TLR4) expression in NSCs. Evidence also indicates that TLR4 is involved in promoting NSC differentiation into astrocytes and neurons during stroke progression [158]. Depletion of circTTC3 was found to significantly decrease neurological scores, brain water content, and cerebral infarction in a middle cerebral artery occlusion/repression stroke model. Moreover, the effect of circTTC3 on stroke and NSC biology has been confirmed to be mediated through the miR-372-3p/TLR4 axis. 

### 4.3. Acbd6

The circRNA Acbd6 is spliced from exons derived from Acbd6 on the positive strand of chromosome 13 (from 73, 239, 821–73, 265, and 813 bp). Virtually no reports have investigated circAcbd6, and few studies have examined the host genes of circAcbd6. However, members of the ACBD family have been reported to play important roles in regulating viral replication, organelle organization, self-renewal of stem cells, and protein acylation [159]. A modular protein in mammalian cells, acyl-coenzyme A binding domain-containing member 6 (Acbd6) is found in the spleen, placenta, and embryonic-like stem cells produced from the placenta, bone marrow, cord blood, and circulating CD34+ progenitors [159,160]. In a recent study, a particular region of the SGZ that harbors a subpopulation of mature NSCs showed a substantial expression of circAcbd6 in neural tissues. Moreover, the finding from our lab shows that the forced expression of circAcbd6 promotes hippocampal NSC differentiation into neurons and even cholinergic neurons [161]. In vivo results suggest that circAcbd6 may greatly enhance learning and memory ability, indicating its potential value in the treatment of AD.

**Table 3:** Effects and mechanisms of various circRNAs in NSCs.

Name	Biologic Function	Mechanism	References
circHIPK2	Inhibit proliferation and differentiation of NSCs	Combine miR-124 and regulate Smox expression	[146]
circRNA TTC3	Inhibit proliferation and differentiation of NSCs	Regulate miR-372-3p/TLR4 axis	[157]
hsa-circ-0002468	Regulate neuronal differentiation	Modulate miR-561/E2F8 axis	[162]
circRNA Acbd6	Promote NSC differentiation into cholinergic neurons	Modulate miR-320-5p-Osbpl2 axis	[161]
circ-0005835	Inhibit NSC proliferation and differentiation to neurons	Sponge miR-576-3p	[163]

## 5. Studies of ncRNAs in Hippocampus-Related Diseases 

### 5.1. Alzheimer’s Disease

Alzheimer’s disease (AD), a progressive neurodegenerative disease involving multiple damaged brain regions (including the hippocampus), is the main cause of dementia in older people globally. It is characterized by the deposition of Aβ plaques, neurofibrillary tangles, and chronic inflammation, thus leading to a loss of neurons; this loss is closely associated with the accumulation of Aβ and the hyperphosphorylation of Tau [164,165,166]. Many studies in patients with AD have reported many upregulated miRNAs in the brain (including miRNA-7, miRNA-9, miRNA-34a, miRNA-125b, miRNA-146a, and miRNA-155) [167,168]. According to a recent study in *Nature Neuroscience*, a substantial correlation exists between AD characteristics and circHOMER1, circMAN2A1, and circKCNN2. circHOMER1, circKCNN2, and other circRNAs were discovered in all four meta-analyses; two quantitative AD characteristics have also been associated with these eight circRNAs (circDNAJC6, circFMN1, circDOCK1, circERBIN, circHOMER1, circKCNN2, circMAN2A1, and circST18) [169,170]. Idda et al. analyzed and reported the abnormal expression of lncRNA in AD and found that BACE1-AS, 51A, and NAT-Rad18 are important ncRNAs that are involved in the onset of AD [171].

In view of these abnormally expressed ncRNAs and important target genes, anti-miRNA strategies, anti-NF-kB strategies, and other strategies for the treatment of AD have been proposed. RNA oligonucleotides, which have recently been shown to potently suppress miRNAs, are being developed as anti-miRNA drugs, and techniques using viral vector delivery systems have been found to maintain miRNA-associated homeostasis in brain tissue [172]. The ncRNAs involved in AD are summarized in Table 4.

### 5.2. Epilepsy

Epilepsy, a common chronic disease affecting the nervous system, is characterized by recurrent spontaneous seizures and affects approximately 1% of the global population. The hippocampus is an important area in relation to the mediation of epileptic discharges [217]. Although great progress has been made in the development of antiepileptic drugs, some patients with epilepsy are not sensitive to drug treatment, and many patients have adverse reactions to antiepileptic drugs. Epilepsy is attributed to an imbalance between inhibitory and excitatory neurons in the CNS that leads to the “abnormal discharge” of neurons [218].

In many cases, epilepsy occurs alongside a neurodegenerative disorder, such as AD. Some patients with epilepsy experience memory impairment, whereas 16% of patients with AD develop epilepsy during severe dementia [219]. Post-stroke epilepsy can occur shortly after an ischemic attack or later, and acute ischemia can increase the concentrations of extracellular glutamate, thereby leading to periodic epileptiform discharge in neurons [220,221,222].

More and more ncRNAs have been found to be closely involved in the genesis or pathophysiological processes of epilepsy, exhibiting the value of biomarkers for diagnosis and prognosis [223,224]. Taking miRNA as an example, accumulating evidence indicates that the miRNA landscape is dysregulated in epilepsy, igniting prospects for the treatment of epilepsy [225,226]. The strategy aiming at miRNA involves administration of chemically engineered antisense oligonucleotides (ASOs, mimics or antagomirs) targeting the given miRNAs, which would thus be reproduced or inhibited. For example, miRNA-124 is a very small important RNA molecule whose expression is highest in the hippocampus. During the onset of epilepsy, by the addition of an activator, levels of miRNA-124 may increase, thus regulating CREB1 and other genes and consequently decreasing the degree of seizures and preventing excessive neuronal apoptosis [227,228]. Jimenez-Mateoset et al. found that in vivo administration of antagomirs targeting miR-134 could upregulate spine volume in the hippocampus and attenuate the degree of seizures in epileptic mice [229]. These studies provide evidence to support the use of specific ASOs targeting miRNAs or other ncRNAs for anticonvulsant effects.

### 5.3. Stroke and I/R Injury

The hippocampus is usually the victim in the process of stroke and ischemia reperfusion (I/R) injuries, which are often followed by episodic memory impairments. Increasing evidence indicates that ncRNAs exert crucial roles in the pathophysiological processes of stroke or I/R injury, including neuroinflammation, oxidative stress, excitotoxicity, and apoptosis, as summarized in Table 5. With the increasing incidence of stroke and I/R injury, studies examining the related pathogenesis have found that regulation of ncRNAs promotes the reversal of these diseases. For instance, the lncRNA MEG3 inhibits neuronal death and neurological problems in mice with ischemic stroke through the MEG3/miR-378/Grb2 axis and inhibits neuronal autophagy and neurodegeneration by silencing Grb2. In vitro studies have revealed that MEG3 specifically binds miR-378 and increases Grb2 expression, thus inhibiting the activation of the Akt/mTOR pathway and protecting neurons against autophagy and neurological damage [230]. Studies on animals showed that using tailored exosomes enriched with miR-17-92 clusters led to improvement of neurological function and enhancements of neurogenesis during brain stroke via activation of the PI3K/AKT/mTOR signaling pathway [231]. Intravenous administration of an antagomir against miR-15a/16-1 significantly alleviated ischemic damage via upregulation of antiapoptotic proteins and inhibition of proinflammatory molecules [232]. lncRNAs and circRNAs were also reported to play critical roles in the progression of ischemic stroke, thus likewise acting as potential therapeutic targets for stroke. Therefore, treatments which target ncRNAs or make use of ncRNA molecules might help to improve the prognosis of stroke and I/R injury.

## 6. Limitations and Prospects

Current research has utilized the characteristics and advantages of ncRNAs (especially miRNAs and circRNAs) as new biological markers to screen for several diseases. These ncRNAs have major roles in disease initiation and progression and great value in disease diagnosis, treatment, and prognostication. Interfering with the regulation of ncRNAs can help control disease progression; therefore, regulation of the expression of key ncRNAs may provide hope for disease prevention and treatment and the identification of targets for new drug development. At present, the future of ncRNA research is promising. Currently, in the treatment of neurological illnesses, ncRNAs can be regulated through a range of physical techniques, such as cranial magnetic resonance and electroacupuncture. However, recent studies have shown that the development of NSCs is a precisely controlled process whose influencing factors are not regulated by single signaling pathways but are instead closely associated with a variety of different cellular signaling pathways and cellular electrophysiological characteristics. The regulatory network is complex, involving chromatin remodeling, histone modification, DNA methylation, X chromosome inactivation, and regulation of ncRNA at the translational level. It is also associated with changes in the extracellular microenvironment. Recent studies have shown that ncRNAs play important roles in the differentiation of NSCs. However, several limitations must be noted. Research on the effects of ncRNAs on NSC differentiation has been relatively simple and limited to examining the effect of a certain type of ncRNA on NSC differentiation. The specific mechanisms remain to be fully elucidated, and some influencing factors remain hypothetical. In addition, few reports have described the formation of common regulatory networks among ncRNAs, mRNAs, and the microenvironment affecting the differentiation of NSCs. Furthermore, the effects of ncRNAs on the differentiation of NSCs involve multiple molecules. Although in recent years lncRNAs have been confirmed to act as miRNA sponges, few studies have assessed the effects of ncRNAs on the nervous system. Moreover, the construction of lncRNA-miRNA-mRNA regulatory networks is imperfect, and further research in this field is urgently needed. Nonetheless, ncRNAs are important regulators affecting the differentiation of NSCs and may enable the development of potentially valuable novel treatments for nervous system diseases.

## Figures and Tables

**Table 4:** Summary of ncRNAs involved in AD.

**Name**	**Target Protein/Gene/Process**	**References**
miRNAs		
miR-107	BACE1	[173,174]
miR-29a/b-1	BACE1	[175,176]
miR-9	BACE1	[176,177]
miR-188-3p	BACE1	[178]
miR-339-5p	BACE1	[179]
miR-195	BACE1	[180,181]
miR-186	BACE1	[182]
miR-27a-3p	Presenilin-1	[183]
miRNA-34a	Tau, γ-secretase	[184,185]
miRs-24, miR-186, miR-455	Nicastrin	[186]
miR-144	ADAM10	[187,188]
miR-125b, miR-146a	Tetraspanin12	[189,190,191]
miR-101	APP, Tau	[192,193,194]
miR-16	APP	[195,196]
miR-147, miR-20a	APP	[197]
miR-124	PTBP1, BACE1	[198,199,200]
miR-132, miR-212	GSK3, Tau, INOS	[201,202]
miR-15, miR-16	ERK1, CDK5R1/p35	[203,204]
miR-33	ABCA1 and APOE lipidation	[205,206]
lncRNA		
BACE1-AS	BACE1	[207,208]
lncRNA 51A	SORL1	[209,210]
BDNF-AS (NAT)	BDNF	[209,210,211]
LRP1-AS	LRP1	[210,212]
NAT-Rad18	RAD18	[210,213]
AD-linc1, AD-linc2	Unknown	[210,214]
EBF3-AS (NAT)	Nuclear processes	[210,214]
HAO2-AS (NAT)	Nuclear processes	[210,214]
circRNA		
ciRS-7	Sponge for miR-7 to regulate UBE2A	[215,216]

**Table 5:** Summary of ncRNAs involved in stroke and I/R injury.

**Name**	**Putative Regulators and Targets**	**Signaling Pathways**	**Functions**	**Ref.**
miRNA				
miR-146a	IL-1β, IRAK1, TNF-α, IL-6	NF-κB	Aggravate cerebral I/R injury	[233]
miR-155-5p	DUSP14	NF-κB, MAPK	Accelerate cerebral I/R injury	[234]
miR-125b	p53	Bax/Cytochrome C/Caspase-3	Protect cerebral I/R injury	[235]
miR-25	Bax, Caspase-3, Bcl-2	Fas/FasL	Inhibit cerebral I/R injury-induced apoptosis	[236]
miR-22	Ang-1, VEGF	PI3K/AKT	Protect and promote angiogenesis in cerebral I/R	[237]
lncRNA				
H19	Beclin1, DUSP5, LC3I, L3II, P62	ERK1/2	Onset of I/R-induced inflammation	[238]
MEG3	BDNF, bFGF, NGF	Wnt/β-catenin	Enhance OGD/R-induced pyroptosis and inflammation during cerebral I/R injury	[239]
Oprm1	GATA3	NF-κB	Alleviate apoptosis in cerebral I/R injury	[240]
MALAT1	miR-145, AQP4		Accelerate cerebral I/R injury	[241]
circRNA				
circTTC3	TLR4		Accelerate cerebral I/R injury	[157]

## Data Availability

No data support in publicly archived datasets.
